# Contactless Video-Based Heart Rate Monitoring of a Resting and an Anesthetized Pig

**DOI:** 10.3390/ani11020442

**Published:** 2021-02-08

**Authors:** Meiqing Wang, Ali Youssef, Mona Larsen, Jean-Loup Rault, Daniel Berckmans, Jeremy N. Marchant-Forde, Joerg Hartung, André Bleich, Mingzhou Lu, Tomas Norton

**Affiliations:** 1 Faculty of Bioscience Engineering, Katholieke Universiteit Leuven (KU LEUVEN), 3001 Leuven, Belgium; meiqing.wang@kuleuven.be (M.W.); ali.youssef@kuleuven.be (A.Y.); monalilianvestbjerg.larsen@kuleuven.be (M.L.); daniel.berckmans@kuleuven.be (D.B.); 2 Institute of Animal Welfare Science (ITT), University of Veterinary Medicine (Vetmeduni) Vienna, A-1210 Vienna, Austria; Jean-Loup.Rault@vetmeduni.ac.at; 3 USDA-ARS, Livestock Behaviour Research Unit, West Lafayette, IN 47907, USA; Jeremy.marchant-forde@usda.gov; 4 Institute of Animal Hygiene, Animal Welfare and Farm Animal Behaviour, University of Veterinary Medicine Hannover, 30625 Hannover, Germany; joerg.hartung@tiho-hannover.de; 5 Institute for Laboratory Animal Science and Central Animal Facility, Hannover Medical School, 30625 Hannover, Germany; Bleich.Andre@mh-hannover.de; 6 College of Engineering/Jiangsu Province Engineering Lab for Modern Facility Agriculture Technology & Equipment, Nanjing Agricultural University, Nanjing 210031, China; lmz@njau.edu.cn

**Keywords:** heart rate monitoring, contactless video, single-channel signal, short-time Fourier transform, pig health and welfare

## Abstract

**Simple Summary:**

Contactless physiological monitoring can be important for animal health and well-being. The current study investigated whether heart rate in pigs can be extracted automatically from videos without disturbing the pig and showed that this was possible with 4.69 beats per minute in mean absolute error. The study also tested different body regions and found that the abdomen was a better region to measure heart rate from videos compared to the front leg or the neck. However, future studies are needed that include videos with different light conditions, different housing systems and multiple pigs to enable real-time on-farm monitoring of heart rate from videos.

**Abstract:**

Heart rate (HR) is a vital bio-signal that is relatively easy to monitor with contact sensors and is related to a living organism’s state of health, stress and well-being. The objective of this study was to develop an algorithm to extract HR (in beats per minute) of an anesthetized and a resting pig from raw video data as a first step towards continuous monitoring of health and welfare of pigs. Data were obtained from two experiments, wherein the pigs were video recorded whilst wearing an electrocardiography (ECG) monitoring system as gold standard (GS). In order to develop the algorithm, this study used a bandpass filter to remove noise. Then, a short-time Fourier transform (STFT) method was tested by evaluating different window sizes and window functions to accurately identify the HR. The resulting algorithm was first tested on videos of an anesthetized pig that maintained a relatively constant HR. The GS HR measurements for the anesthetized pig had a mean value of 71.76 bpm and standard deviation (SD) of 3.57 bpm. The developed algorithm had 2.33 bpm in mean absolute error (MAE), 3.09 bpm in root mean square error (RMSE) and 67% in HR estimation error below 3.5 bpm (PE_3.5_). The sensitivity of the algorithm was then tested on the video of a non-anaesthetized resting pig, as an animal in this state has more fluctuations in HR than an anaesthetized pig, while motion artefacts are still minimized due to resting. The GS HR measurements for the resting pig had a mean value of 161.43 bpm and SD of 10.11 bpm. The video-extracted HR showed a performance of 4.69 bpm in MAE, 6.43 bpm in RMSE and 57% in PE_3.5_. The results showed that HR monitoring using only the green channel of the video signal was better than using three color channels, which reduces computing complexity. By comparing different regions of interest (ROI), the region around the abdomen was found physiologically better than the face and front leg parts. In summary, the developed algorithm based on video data has potential to be used for contactless HR measurement and may be applied on resting pigs for real-time monitoring of their health and welfare status, which is of significant interest for veterinarians and farmers.

## 1. Introduction

Cardiac activity variables have been widely used in animal health and animal welfare research. For instance, heart rate (HR) brings valuable information in relation to an animal’s disease status, physiological functioning, psychological stress and in assessing their individual characteristics, e.g., temperament and coping strategies [1]. Farm animals such as pigs may encounter many stressors during their lifetime, and HR monitoring has proven to be a useful technology to assess their response in many cases. For example, monitoring the HR of sows showed that their reproductive performance was impaired when they experienced stress [2]. Other research has shown that HR can potentially be a valuable measure for stress resilience, e.g., a decrease in HR was linked with a counteraction of the sympathetic overreaction caused by the chronic stress of tethering in sows [3]. Therefore, monitoring HR of pigs can provide information on how to maintain optimal conditions for production performance and animal welfare.

In previous studies, HR of animals was monitored using two types of technologies, namely implantable transmitters and external body-mounted sensors [1]. Although implantable transmitters provide accurate data, their use is associated with several critical drawbacks, e.g., initial implantation surgery [4], influence on normal circadian rhythms [5], potential distress and discomfort caused by the implanted device itself and the limited battery life of the transmitter [6]. For body-mounted sensors, electrocardiography (ECG) has been used to monitor the HR of dogs [7] and bats [8], and photoplethysmography (PPG) [9] has previously been used for HR monitoring in pigs. However, ECG and PPG signals may be disrupted by the poor contact between the electrodes and the skin and the movement/removal of the device/belt caused by the pig itself or by its conspecifics [1]. Moreover, devices such as a belt are not applicable in field situations where pigs are typically housed together with conspecifics. Compared to these two technologies, a contactless method based on video analysis could have significant potential in research and farm applications involving HR monitoring, as it obviates the need to fit/implant sensors on/in animals or any other manipulation of the animal, avoiding potential disruptions for sample collection. Therefore, a contactless HR monitoring system based on video analysis is a non-invasive and non-intrusive method that could help assessing pig health and welfare.

Presently, HR extraction based on different video analysis techniques extract the subtle changes (color or motion) caused by the pulsatile activity of the beating heart. Some of the recently developed algorithms are based on blind source separation (BSS), i.e., separation of the source signals from a set of mixed signals without prior information about the source signals [10,11], such as independent component analysis (ICA) [10] or principle component analysis (PCA) [11]. Using the BSS approach, the red, green and blue(RGB) traces are decomposed into the three independent sources of signals. After that, the highest power of the spectrum of the component containing the heart signal [10] or the frequency of the principal component that most closely resembles the heart signal [11] is considered as the HR frequency/signal. Other algorithms able to extract HR-related information from video signals include techniques such as combining three channels under different proportions [12,13] or processing each pixel independently by Eulerian video magnification (EVM) [14,15]. It should be noticed that both kinds of methods use three channels to extract HR, and because of that, they need three filters to remove noise and three times the computer memory to store the signal during the process of computing. However, to achieve accurate continuous monitoring of HR in practical environments, the implemented algorithm must be accurate while having a computational footprint as low as possible to run on embedded systems. To reduce the computing complexity, this study attempts to use a single color channel to monitor a pig’s HR.

Recently, the video-based HR monitoring approach has been explored in animal research including cattle [16], primates [17] and rodents [18]. Video-based monitoring of pigs has become popular on farms, mainly for behavior recording [19,20,21,22,23]. There is an opportunity to monitor other bio-signals, extracting greater value for the users of video technology. Although some studies have already analyzed HR of pigs from video data, a limited number achieved it continuously [24,25,26]. To our knowledge, only the study by Addison et al. [27] could continuously monitor the HR using video technology of anesthetized pigs during acute hypoxia. However, the algorithm used in that research was developed based on three channels, which could be improved in terms of computing complexity.

Another vital aspect of accurate monitoring of HR is the identification of a region of interest (ROI). The subtle blood flow changes caused by the heart beats have to be detected from continuous frames and, thus, an adequate body part of the animal has to be assessed. Regions with large capillaries near the skin surface and low hair covering can be chosen as an ROI. In this sense, domestic pigs present a large area of exposed skin with low hair coverage, and commercially housed pigs spend about 60–80% of the daily life resting [28,29], facilitating the monitoring of their HR. On the other hand, pigs are known for their high level of backfat and thickness of epidermis [30], which make changes in blood flow less visible at skin level. Previous studies have used the area around the neck as the ROI [25,26]; however, no studies have explored the suitability of other body regions. Therefore, selecting the ROI was a key aspect explored in this study, as different body parts may offer different sensitivities for HR monitoring.

The main goal of the present work was to develop a video-based HR monitoring algorithm for pigs with minimum computational burden for real-time applications. The specific objectives were to:

1. Investigate the combination of bandpass filtering and short-time Fourier transform (STFT) with sliding windows for extracting HR from noisy input data in a continuous fashion;
2. Explore different regions of interests (ROI) from different anatomical parts of the pig’s body to find the most suitable ROI for signal extraction of cardiac activity;
3. Optimize the different heart rate extraction processing steps to minimize the computational complexity of the algorithm for implementation of real-time monitoring applications.

## 2. Materials and Methods

### 2.1. Experimental Setup

The datasets used in the analysis were obtained from two different experimental set-ups. In the first experiment, video recordings of one anaesthetized pig was made and its data used to develop the algorithm, as the pig had very limited movement (breathing) with no motion artefacts. In the second experiment, a non-anaesthetized resting pig was video recorded. The pig from the second experiment presented larger variations in HR, and its data were used to test the developed algorithm. All the raw videos were stored on an exchangeable, external 4 TB hard drive (Seagate, Cupertino, CA, USA). The authors are aware of the limited number of animals and variation included in the data and what limitations this has for the interpretation of the developed algorithms. However, this was evaluated as acceptable, as the study presents the initial work on a new method for HR monitoring.

#### 2.1.1. Experiment on the Anesthetized Pig

**Figure 1:**
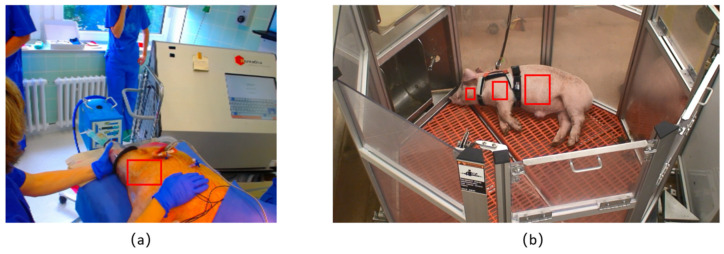
(**a**) Experiment set-up of the anesthetized pig; (**b**) experiment set-up of the resting pig. The red rectangles represent regions of interest chosen to investigate for heart rate monitoring.

The first dataset included one anaesthetized two-year-old Göttinger Minipig (Figure 1a), weighing 30.2 kg. Zoletil (Tiletamin and Zolazepam) and isoflurane were used to anesthetize the pig. The experiment was conducted under ambient light. The webcam (C920 HD PRO, Logitech, Taiwan, China) was positioned above the pig towards the neck and the side face of the pig at a distance of 0.84 m with about 45 degrees angle. The resolution of the video was 640 × 480 pixels and the frame rate was 30 fps. The video chosen for the algorithm development was 180 seconds long. As a gold standard (GS), the reference HR was collected using an electrocardiogram (BEAM EKG-/Loop- Eventrekorder; I.E.M. GmbH, Stolberg, Deutschland), and it measured the HR (200 Hz, 0,1–75 Hz) directly from the skin region above the heart.

#### 2.1.2. Experiment on the Resting Pig

The second dataset included one resting, individually housed Large White × York pig weighing 20 kg (Figure 1b). The experiment was conducted at Purdue University, West Lafayette, IN, USA. The pig was placed in a PigTurn^TM^ (West Lafayette, IN, USA) experimental pen with enough space for an individual animal (1.12 m^2^) under ambient light. The animal wore a wearable sensor (Zephyr BioHarness-3) in order to measure its movement with a 3D accelerometer (100 Hz, ± 16 G) and its HR (1 Hz, 25–240 bpm ±1 bpm accuracy), which was used as the GS. Video recordings of the pig were made during the whole experiment using a Sony HandyCam HDR-SR5 camcorder. The camera was positioned on a Manfrotto Autopole at a height of 2.5 and 2 m from the center of the pen. The resolution of the video was 1440 × 1080 pixels, and the frame rate was 30 fps. One period of the video of about 450 s was chosen and used to test the algorithm, as the pig during this period was constantly resting with minimal movements. 

### 2.2. Algorithm

**Figure 2:**
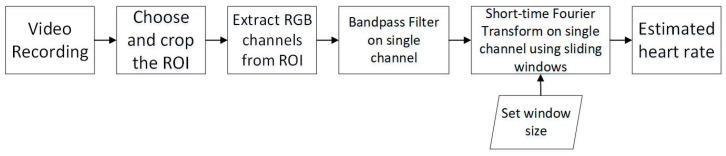
Flow chart representation of the different processing steps for heart rate extraction based on video data.

The different steps taken in the HR extraction algorithm are represented in Figure 2. The algorithm was developed and tested on the raw videos. All analyses and calculations were performed in the MATLAB (MathWorks, US) environment. All the analysis were conducted on Windows 10 software with a Inter(R) i7-8650U CPU and 16GB RAM.

#### 2.2.1. Region of Interest Selection

The first challenge in developing the algorithm was to identify the ROI for pigs. Subtle changes in the video signal are used to extract heart rate. Thus, in human applications, places with large capillaries near the skin surface and low hair covering are frequently chosen as the ROI [31]. The skin around the neck was, thus, chosen from the anaesthetized pig for developing the algorithm (red rectangle in Figure 1a). Further, pigs have large subscapular artery and median sacral artery that provide extensive flow to their front legs and abdomen [32]. In order to see which part was most sensitive to fluctuating HR extraction, body areas around the neck, front legs and abdomen were chosen as potential ROIs (red rectangles in Figure 1b) from the video of the resting pig. To develop the algorithm, the color variations through all video frames for all pixels of the ROI are needed. The pre-processing step first extracted RGB values for all pixels of the ROI from every frame of the video and stored them in a multidimensional matrix. Then, the average values were individually computed in red, green and blue channels of each frame. As a result, the colors in the ROI for each frame were represented by the corresponding average RGB values, and three time series based on three channels were derived.

#### 2.2.2. Noise Removal

The finite impulse response (FIR) bandpass filter has been widely used in suppressing frequency components outside the HR bandwidth [17,25]. Compared to an infinite impulse response (IIR) filter, it is less susceptible to finite bit precision effects [33]. In this study, an FIR bandpass filter was introduced to obtain HR-related ranges. The cut-off frequencies are chosen based on the expected physiological HR range [34]. Frequency components outside of the HR bandwidth 30–360 bpm are supposed to be suppressed. In our case, the frequency range of the bandpass filter was set to 0.5–3 Hz. 

#### 2.2.3. Heart Rate Extraction

The short-time Fourier transform (STFT) is a variant of the classic Fourier transform, where a window function is convolved with the original signal to only transform a short part of the signal into the frequency domain. Hence, a simultaneous representation in time and frequency can be achieved. The length and shape of the window function can control the time and frequency resolution. Due to the uncertainty principle, there is always a trade-off between time and frequency resolution. If the window size is too large, almost all the frequency information will be captured, which is similar with using fast Fourier transform (FFT) over a long window. In this case, the time resolution would be lost and vice versa for small windows. In order to find the optimum setting, we tested different window sizes and window functions. Specifically, the signal was first split into pieces by the windows. Then, a window function was adopted to time the data points in every piece. After that, Fourier transformation was performed in each piece, and the amplitude of each frequency was computed. Finally, the HR was computed based on the amplitude and the predefined frequency range (based on the range of the GS HR). The frequency that holds the highest amplitude is considered as the HR frequency. Note that the overlap between two continuous windows was set to three quarters of the window, and f1 and f2 were 0.6 and 6 Hz, respectively. The computing details were as follows:
**Algorithms 1** Computation details of HR extraction**Input:** single colour signal $S=s_{i}\left(i=1,2,\ldots N\right)$; sampling rate r; window size w; window function wf; overlap window size wn (here wn=3*w/4); minimum frequency f1 and maximum frequency f2.**Output:** Estimated heart rate hr1:min_hr_freq=1+f1*w/r2:max_hr_freq=1+f2*w/r3:**For** i in [1, 2, …, K, K= ⌊N/(w-wn) ⌋ ] **do**4:${\mathrm{win}}_{i}=(i-1)*(w-\mathrm{wn})+1$5:**end for**6:**For** k in [1,2,…K] **do**7:${\mathrm{fwin}}_{k}{=(s}_{p}{:s}_{q})*\mathrm{wf},\;$${\mathrm{where}\;p=\mathrm{win}}_{i}{\;\mathrm{and}\;q=\mathrm{win}}_{i}+\left(w-\mathrm{wn}\right)-1$8:$A_{k}=\mathrm{abs}\left(\mathrm{fft}\left({\mathrm{fwin}}_{k}\right)/w\right)$9:$A_{k}{=A}_{k}\left(\min\_\mathrm{hr}\_\mathrm{freq}:\max\_\mathrm{hr}\_\mathrm{freq}\right)$10:${\mathrm{HRFreqIndex}}_{k}=\max\left(A_{k}\right);$11:${\mathrm{hr}}_{k}=\left(60*\frac{r}{w}\right){*(\mathrm{HRFreqIndex}}_{k}+\min\_\mathrm{hr}\_\mathrm{freq}-2)$12:**end for**

#### 2.2.4. Validation

To investigate the quality of HR estimation, mean absolute error (MAE), root-mean-square error (RMSE) and percentage of HR error below 3.5 (PE_3.5_) beats per minute (bpm) were used to make the comparison with the GS HR. Assume that the estimated and reference HR are $p_{1}$ and $p_{2}$, respectively, then MAE, RMSE and PE_3.5_ are given by:

(1) $$\mathrm{MAE}=\;\frac{1}{N}\underset{i}{\sum }\left|p_{1}-p_{2}\right|$$

(2) $$\mathrm{RMSE}=\;\frac{1}{N}\sqrt{\underset{i}{\sum }{\left(p_{1}-p_{2}\right)}^{2}}$$

(3) $$PE_{3.5}=\;\frac{1}{N}\{i:\left|p_{1}-p_{2}\right|<3.5\;BPM\}$$

where *N* is the total number of windows, and *i* is the *i*-th window. 

#### 2.2.5. Channel Selection

The core of extracting HR from the video is to extract effective information from RGB channels. As different color channels contain different levels of information regarding HR [17], first, three single channels (R, G and B) were tested individually by using STFT to extract HR. Then, the extracted HR was compared with the GS HR by computing MAE, RMSE and PE_3.5_. Finally, the channel that performed best in validation was selected as the one used in monitoring.

#### 2.2.6. Algorithm Comparison

After channel selection, only one channel was used in the developed algorithm for extracting the HR. Less computation is needed in this way. However, the accuracy of this study was also compared to the algorithms using three channels that included combining three channels in different proportions [12,13,35,36] and blind source separation method based on ICA [10].

In previous studies [12,13,35,36] of extracting HR from video signal, three-channel signals were combined in four different proportions: green-red difference (GRD), adaptive green-red difference (aGRD), chrominance-based method (CHROM), plane-orthogonal-to-skin (POS), and they were defined as follows. Assume r, g, b are the color signal of the three channels, then:

GRD = g − r(4)

(5) $$\mathrm{POS}\;=g-b+\frac{\sigma_{x}^{L}}{\sigma_{y}^{L}}\;\left(g-b-2r\right)$$

where $\sigma_{x}^{L}$ and $\sigma_{y}^{L}$ are *L*-point running standard deviations of *x* = g − b and *y* = g + b − 2r, respectively;

(6) $$\mathrm{CHROM}=0.77r-0.51g-\frac{\sigma_{x}^{L}}{\sigma_{y}^{L}}\left(0.77r+0.51g-0.77b\right)$$

where $\sigma_{x}^{L}$ and $\sigma_{y}^{L}$ are *L*-point running standard deviations of *x* = 0.77r − 0.51g and *y* = 0.77r + 0.51g − 0.77b, respectively;

(7) $$a\mathrm{GRD}=\left|\left|c_{0}\right|\right|\left(\frac{g}{g_{0}}-\frac{r}{r_{0}}\right)$$

where $g_{0}$ and $r_{0}$ are the average of g and r channel of all pixels in ROI,

(8) $$c_{0}=\sqrt{r_{0}^{2}+g_{0}^{2}+b_{0}^{2}}$$

**Figure 3:**
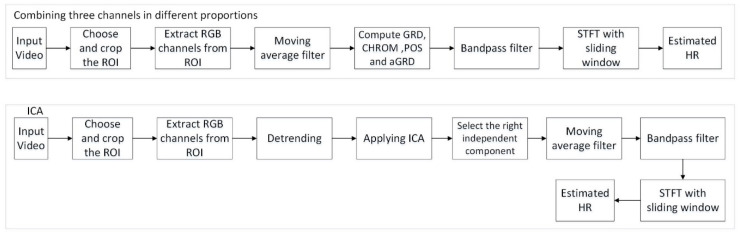
The workflow of combining three channels and independent component analysis (ICA).

Besides the above channel combination methods, blind source separation based on ICA [10] were also used for comparison. To select the best component when doing ICA, fast Fourier transform (FFT) was applied on the output sources and chose the one with the highest peak. Their workflow is showed in Figure 3.

## 3. Results and Discussion

**Figure 4:**
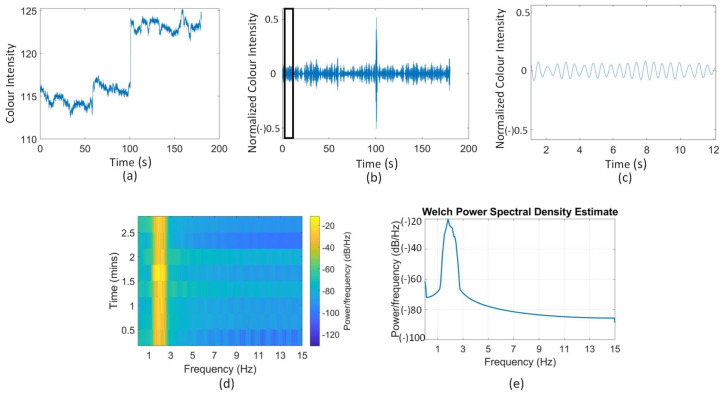
(**a**) The original G channel signal of the anaesthetized pig plotted in time-domain; (**b**) the filtered G channel signal; (**c**) a zoom-in view of the filtered signal; (**d**) time-frequency spectrogram; (**e**) the Welch power spectrum density (PSD) estimate.

**Figure 5:**
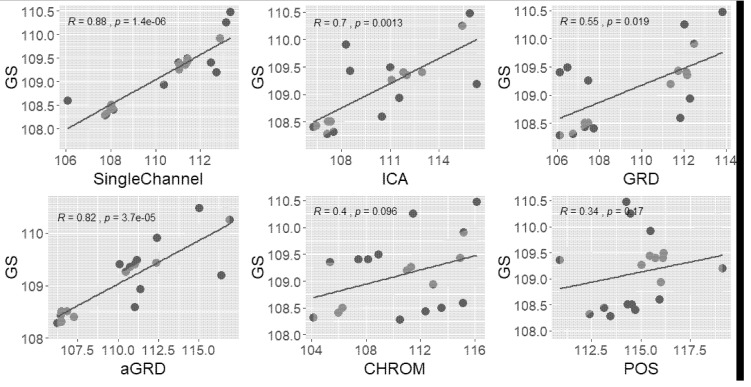
Correlation between monitoring of HR on the anaesthetized pig using the gold standard (GS) vs. using a single channel, combination of the three channels and ICA.

**Figure 6:**
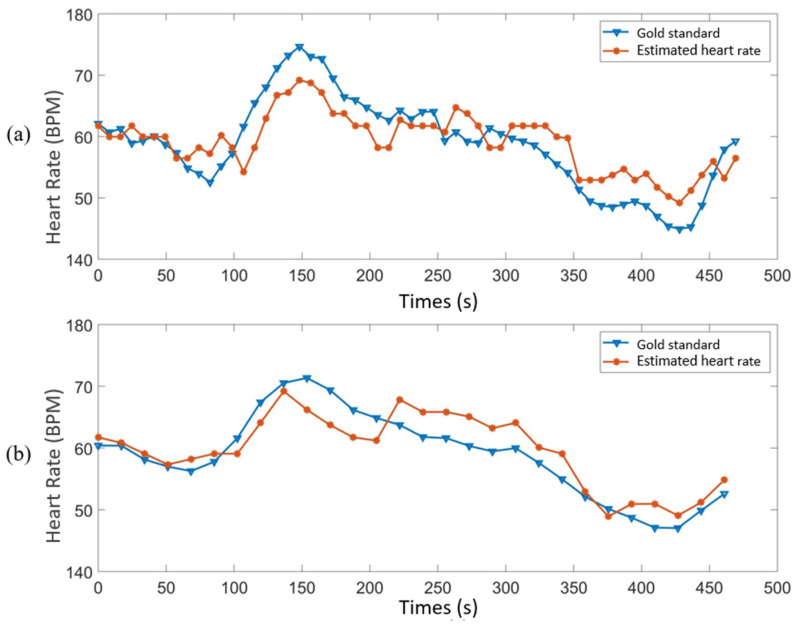
The comparison between gold standard and monitoring results from the skin surface of the abdomen of the resting pig: (**a**) window size 68.27 s; (**b**) window size 34.14 s.

The video of the anaesthetized pig was used for choosing the channel and comparing with other algorithms; the results are shown in Figure 4 and Figure 5 and Table 1 and Table 2. The video from the resting pig was used to further test different ROI, and the related results can be found in Table 3 and Table 4 and Figure 6. 

Figure 4 shows the noise removal and spectral information of the anaesthetized pig. Specifically, Figure 4a–c illustrates the original color signal (green channel), the filtered signal and a zoom-in view of the filtered signal plotted in the time domain, respectively. The filtered signal is clearer than the original one and has regular patterns related to HR. Figure 4d is the spectrogram generated from the filtered signal, showing that the noise was removed effectively. Figure 4e shows the Welch power spectrum density (PSD) estimate where the cut-off frequency was set to 3 Hz. We can see that in Figure 4f, the energy is mostly around 1.5 to 2.5 Hz with a center frequency around 1.8 Hz, which falls in the expected HR range of the anaesthetized pig.

Table 1 shows the validation results of MAE, RMSE and PE_3.5_ from R, G and B channels of the anaesthetized pig. Based on a lower MAE, RMSE and a higher PE_3.5_, the G channel is better than the other two channels. Hence, the G channel is, in the current study, considered the best for HR extraction in pigs and is the channel used in further analysis. In order to find the best HR estimation of the anaesthetized pig, different window sizes and window functions were tested on the three RGB channels. Note that the overlap between two continuous windows was set to three quarters of the window and the GS HR used the same window and overlap sizes as the video data. Four window sizes were tested on the video of the anesthetized pig: 8.53, 17.07, 34.13 and 68.27 s, representing the number of data points 256, 512, 1024 and 2048, respectively, used in the windows. Different window functions (*rect, hamming, hanning and blackman*) were also tested. By comparing the MAE, RMSE and PE_3.5_ from different window sizes in Table 1, we can see that the lowest MAE and RMSE were obtained with window size 68.27 s, but the PE_3.5_ of this window was not the highest. This might result from some extreme large or small HR estimations when using window size 68.27 s. Thus, considering the highest PE_3.5_ and relatively low MAE and RMSE, the best window size for the anesthetized pig’s HR extraction was 34.13 s. Further, by comparing the MAE, RMSE and PE_3.5_ from different window functions of window size of 34.13 and 68.27 s in the G channel, *hamming* and *hanning* worked better than *rect* and *blackman*, and there was not much difference between *hamming* and *hanning*. If we compare the MAE, RMSE and PE_3.5_ from window size 8.53 and 17.07 s, we can see that *rect* performed better than *hamming* and *hanning*; this may be caused by the small data sample size. More experiments should be conducted in future to further investigate this matter. For further analysis, only window function *hamming* will be used.

Regarding the extraction accuracy and computing complexity of using the single G channel, the current study made a comparison with three-channel-based methods that includes combining three channels in different proportions of GRD, aGRD, CHROM and POS [12,13,35,36]. The video used for comparison was the one extracted from the anaesthetized pig. The pre-processing of all the methods were the same including bandpass filter range (shown in Figure 3), window size (34.13 s), overlapping style (3/4 of the window size) and window function (*hamming*). Table 2 shows the MAE, RMSE and PE_3.5_ as well as computation time regarding the comparison of the different methods. Note that the computation time included the reading of the video and all the processing time. It can be concluded that in the current study, the result obtained by the single G channel is better than for the other methods. Figure 5 shows the correlation between the test results from GS HR and the different methods. Note that R and p in Figure 5 indicate the correlation and p value respectively. The dark dotes represent HR numbers. Besides Figure 5 also gives the 95% confidence interval. We can observe that using the single G channel had the highest correlation with the GS HR. It is interesting that the second best result was from aGRD, which uses the signal of G and R channels, whereas other methods using three channels performed worse. This suggests that using more channels would not yield the best result, while the G channel seems the most effective for extracting HR from videos of pigs. This might result from the fact that the G channel has a higher absorption of hemoglobin. 

The resting pig has larger variations in HR than the anaesthetized pig, which was used to test the sensitivity of the algorithm, using only the G channel, the 34.13 and 68.27 s window sizes and *hamming* window based on the results described above.

Table 3 shows the estimated results from different ROIs of the resting pig, which includes the face, front leg and abdomen. In order to find out which ROI is physiologically best, the effect of pixel number was excluded with the size of the three ROI all set to 46 × 49 pixels. The lengths of the windows were set to 34.13 and 68.27 s, representing 1024 and 2048 data points used in the windows, respectively. Note that the overlap between two continuous windows was set to three quarters of the window, and the GS HR used the same window and overlap sizes as the video data. Comparing the MAE, RMSE and PE_3.5_ of face, front leg and abdomen in Table 3, we can see that the results obtained from the front leg were the worst, and results from the abdomen were better than from the face, especially using window size 68.27 s. Thus, according to the current study, the region of the abdomen is considered physiologically better than the other two parts for HR extraction from RGB videos. This might result from the fact that the backfat of the pig decreases from the shoulder, where it is the thickest, to the last rib, and the ROI of the abdomen is right at the fat-decreasing part [37]. Additionally, Figure 6a,b illustrates the comparison between the GS HR and the monitoring results from the abdomen by using window size 68.27 and 34.14 s, respectively. It can be seen that the developed algorithm can monitor pig’s HR effectively when the pig is in resting status.

This study continuously monitored the HR of a pig by using the single green channel of a video signal. In order to evaluate the monitoring effectiveness on pigs, the current study made a comparison with other species, and the results can be found in Table 4. We can see that the MAE and PE_3.5_ of pigs are more or less the same as for primates, but worse than for humans. We know that animals have more uncontrollable factors than humans, and in this case, the monitoring of HR in pigs on a non-moving subject can be considered acceptable. 

In the current study, the videos of an anaesthetized and a resting pig were used to develop and to test an algorithm to monitor the HR of pigs. From the evaluation parameters of MAE, RMSE and PE_3.5_ (2.33, 3.09 and 67% on the anesthetized pig and 4.69, 6.43 and 57% on the resting pig, respectively), it can be seen that the proposed algorithm was reliable in the currently used setting. Compared to previous studies using three channels, this work decreased the computing complexity by using a single channel. Additionally, from the comparison with other methods, we can conclude that using a single channel also presents the advantage of enhancing accuracy to extract HR from the video of pigs. These advantages may also have the potential to help in the monitoring of other physiological parameters, e.g., respiration rate [26,38]. The demonstrated technique can be an important forward towards continuous monitoring of health and welfare of animals, not only under experimental conditions, but also on farms in the future. However, the experiments were pilot experiments to test the feasibility of using a single G channel to monitor pig’s HR. The data were limited, and the cut-off frequency was designed to only suit the data. In order to improve the robustness of the presented method, it is still necessary to test different lighting conditions, different environmental conditions, different animal and animal sizes as well as animals in movement and in different postures. Thus, much work still needs to be conducted in the future to have a reliable system to monitor HR in pigs without disturbing the animal. Future work should focus on monitoring the physiological parameters of moving pigs with physical movements included. Although this is a serious challenge, it might be achieved by combining an efficient tracking method with the designed monitoring algorithm. In spite of these challenges, we are convinced that digitalization and artificial intelligence will increasingly play an important role in veterinary medicine and animal farming in the future. Applying the presented novel sensor technology for measuring animal-based indicators in real-time, such as heart rate on pigs, is one of the first steps but can mark a paradigm shift in monitoring health and welfare of farm animals. 

## 4. Conclusions

A FIR band pass filter combined with short-time Fourier transform (STFT) based on a single green channel signal allowed us to successfully monitor the HR of pigs in a contactless way from video. Analyzing MAE, RMSE and PE_3.5_, values of 2.33, 3.09 and 67% were obtained from the video of an anesthetized pig and values of 4.69, 6.43 and 57% from a resting pig. The skin area of the abdomen proved to be the most sensitive body region of the three tested for monitoring the HR of the resting pig. The monitoring results obtained from the single green channel presented higher accuracy and needed less computation time than other methods, including combing three color channels in different proportions (DRG, aGRD, CHROM, POS) and ICA. The experimental results indicate that the developed algorithm based on RGB video analysis was capable of monitoring the HR of pigs under the used conditions. It has the potential to be used for contactless heart rate measurement and may be applied on resting pigs for real-time monitoring of their health and welfare status, which is of significant interest for veterinarians and farmers. 

## Figures and Tables

**Table 1:** Validation results of different window sizes (anaesthetized pig) and the three RGB channels presented as mean absolute error (MAE), root mean square error (RMSE) and percentage of heart rate (HR) error below 3.5 beats per minute when compared to the gold standard.

		R Channel			G Channel			B Channel		
Window Size (s)	Window Function	MAE	RMSE	PE_3.5_	MAE	RMSE	PE_3.5_	MAE	RMSE	PE_3.5_
8.53	*rect*	5.41	6.41	37%	4.66	5.25	22%	6.11	6.94	31%
8.53	*hamming*	5.67	6.62	31%	4.74	5.37	22%	6.45	7.31	30%
8.53	*hanning*	5.85	6.80	30%	4.81	5.46	22%	6.72	7.55	27%
8.53	*blackman*	6.17	7.11	26%	5.17	5.90	20%	6.63	7.49	28%
17.07	*rect*	3.86	5.44	54%	3.11	4.61	54%	6.59	7.66	23%
17.07	*hamming*	4.39	5.94	49%	3.26	4.80	54%	7.55	8.69	23%
17.07	*hanning*	4.75	6.40	46%	3.26	4.80	54%	7.55	8.69	23%
17.07	*blackman*	4.83	6.42	46%	3.43	4.96	51%	7.55	8.52	18%
34.13	*rect*	3.36	4.61	67%	2.62	3.40	67%	6.59	7.84	22%
34.13	*hamming*	3.66	4.76	61%	2.33	3.09	67%	6.35	7.54	28%
34.13	*hanning*	3.66	4.76	61%	2.33	3.09	67%	6.48	7.60	22%
34.13	*blackman*	3.68	4.77	61%	2.33	3.09	67%	6.77	7.88	22%
68.27	*rect*	2.96	3.96	57%	2.03	2.76	59%	7.21	8.19	14%
68.27	*hamming*	3.34	4.30	57%	2.03	2.76	59%	8.21	9.57	14%
68.27	*hanning*	3.34	4.30	57%	2.03	2.76	59%	8.21	9.57	14%
68.27	*blackman*	3.46	4.35	57%	2.03	2.76	59%	6.58	8.45	29%

**Table 2:** Accuracy and computing complexity comparison of different methods performed on the anaesthetized pig and presented as mean absolute error (MAE), root mean square error (RMSE) and percentage of HR error below 3.5 beats per minute when compared to the gold standard.

	G	GRD	aGRD	CHROM	POS	ICA
MAE	2.33	2.79	2.76	4.66	4.32	3.04
RMSE	3.09	3.74	3.64	6.07	5.79	4.12
PE_3.5_	67%	53%	49%	33%	41%	54%

**Table 3:** Evaluation results of different regions of interest (ROIs) of the same size on the resting pig presented as mean absolute error (MAE), root mean square error (RMSE) and percentage of HR error below 3.5 beats per minute when compared to the gold standard.

	Face			Front Leg			Abdomen		
Window Size (s)	MAE	RMSE	PE_3.5_	MAE	RMSE	PE_3.5_	MAE	RMSE	PE_3.5_
34.14	5.48	6.98	45%	7.04	8.47	34%	5.24	7.07	53%
68.27	5.64	6.52	29%	6.27	7.52	32%	4.69	6.43	57%

**Table 4:** Accuracy comparison of different species presented as mean absolute error (MAE), root mean square error (RMSE) and percentage of HR error below 3.5 beats per minute (bpm) when compared to the gold standard.

	MAE	RMSE	PE_3.5_
Pig (current study)	4.69	6.43	57%
Primate [17]	4.4	/	56%
Human [31]	1.99	3.25	87%

## Data Availability

Not applicable.
